# Food Security in the Time of COVID-19 for a Marshallese Community

**DOI:** 10.3390/ijerph20043189

**Published:** 2023-02-11

**Authors:** Marcia A. Shobe, Sophie Hill, Yvette Murphy-Erby, Baqir Fateh, Haixia Wang

**Affiliations:** 1 IDEALS Institute, Division of Diversity, Equity, and Inclusion, University of Arkansas, Fayetteville, AR 72701, USA; 2 Fulbright College of Arts & Sciences, University of Arkansas, Fayetteville, AR 72701, USA

**Keywords:** food security, Marshallese, COVID-19, education, economic strain, functional status

## Abstract

Using an individual and family ecological systems model, we explored food security among a Marshallese cohort in Northwest Arkansas during the COVID-19 pandemic. We hypothesized that Marshallese households were experiencing high rates of food insecurity due to socioeconomic and systemic risk factors. Seventy-one Marshallese adults shared socioeconomic information about their household via an online survey. Descriptive results indicate that 91% of respondents report food insecurity. In terms of systemic barriers, almost half of Marshallese respondents do not have health insurance. Additionally, while most respondents report feeling calm, peaceful, and energetic, paradoxically, 81% report feeling depressed and downhearted at least some of the time. Logistic regression findings suggest that food insecurity is significantly related to education levels and household economic strain. These results are analogous with national findings, whereby non-native households are more likely to experience higher levels of food insecurity, lower rates of education, and higher economic strain than native households. As a collective community, the Marshallese could benefit from culturally responsive individual and family systems approaches for improving educational, social, financial, and health opportunities through workforce development, household income and asset development, and food security initiatives. Additional implications for policy, practice, and research are provided.

## 1. Introduction

Access to healthy food is a basic necessity that millions of households in the U. S. lacked prior to the COVID-19 pandemic [1]. Food insecurity is defined as limited or inconsistent access to nutritious, safe, and culturally acceptable food to sustain healthful living for the household [2,3]. Food insecurity is directly correlated with mental, physical, social, and economic disturbances [4,5]. COVID-19 disproportionately affected food security status for communities of color in the U.S. [5], highlighting the critical need for food and nutrition assistance, particularly for racial and ethnic minority populations.

Northwest Arkansas (NWA) is home to the second largest (12,000–15,000 people) Marshallese community outside of the Marshall Islands [6]. Following the 1986 Compact of Free Association (COFA) agreement between the U.S. and the Marshall Islands, and due to climate change and the lack of employment [7], the first Marshallese relocated to NWA in the early 1980s; thousands more followed seeking employment, access to healthcare, and educational opportunities for themselves and their families.

When resettling, migrants face a multitude of challenges including but not limited to language barriers and cultural differences. Inadequate access to food systems containing the necessary nutritional value to thrive further amplifies the inequitable struggles for refugees [1]. Marshallese community members born in the Marshall Islands are ineligible to enroll in the U.S. Supplemental Nutritional Assistance Program (SNAP), a safety net program previously known as food stamps, that provides benefits for food-insecure U.S. households [8]. As a result, Marshallese individuals and families often go hungry or must rely on other mechanisms to ensure a plentiful diet. Currently, Marshallese community members residing in NWA and other surrounding areas have access to two Marshallese grocery stores [9]. Additionally, while there are various community-based food pantries throughout the NWA area, the majority of them require American citizenship to receive food support [6]. Marshallese community members prefer Pacific Islander diets, yet some of the food brought to local pantries is not sufficient in fulfilling their cultural preferences. In addition, local foods that serve as substitutes for indigenous foods available on the Marshall Islands, such as bread fruit, are often higher in carbohydrates and less healthy. Finally, there is limited knowledge within the Marshallese community, particularly for newly arrived individuals and families, regarding American food preparation. As a result, Marshallese community members are at increased risk of experiencing food insecurity.

The COVID-19 pandemic also amplified cultural and linguistic barriers regarding the adoption of health prevention and mitigation recommendations, with local, state, and national responses to the pandemic often failing marginalized communities. For example, conflicting COVID-19 messages regarding risks, limited COVID-19 testing and information about prevention measures, stigma associated with positive test results, and inconsistent health insurance benefits for non-U.S. citizens, all likely contributed to the disproportionate number of COVID-19 illnesses and deaths in the Marshallese community. In terms of linguistic systemic barriers, only 29% of the NWA Marshallese community is fluent in English, resulting in miscommunications and misunderstandings of prevention and treatment notifications from healthcare authorities [6]. Adding to systemic barriers, the Marshallese community seemed to only trust COVID-19 prevention and treatment information provided by the President of the United States in lieu of local healthcare authority communications. In addition, the Marshallese community shares a collectivist culture, which includes celebrations, food, and regular face-to-face interaction with each other, making social distancing during the pandemic very difficult [10,11].

Thirty percent of the local food manufacturer’s factory workforce is comprised of Marshallese community members [9]. The Centers for Disease Control and Prevention (CDC) noted that because local Marshallese employers failed to increase employee benefits (i.e., sick paid leave) and did not fully implement sufficient social distancing measures, Marshallese community members were disproportionately exposed to and died from the coronavirus [6,12].

Using the ecological perspective and family systems ecological theory as our guiding framework, the purpose of this research study was to examine food security and systemic risk factors related to food insecurity for 71 Marshallese households during the early months of the COVID-19 pandemic. This study is one of the first to explore possible systemic barriers to food security as well as the impact of COVID-19 on food security for the NWA Marshallese community. This is particularly salient since there is minimal information about Marshallese household well-being measures in NWA. Findings are designed to help inform future initiatives that address food security as well as systemic barriers for directly achieving food security and indirectly attaining personal, economic, social, and community well-being for the Marshallese community.

## 2. Literature Review and Theoretical Background

### 2.1. Theoretical Model

Bronfenbrenner’s [13] ecological perspective is the theoretical foundation used in this study to help explain food insecurity for individuals. It supports the family ecological systems model [14], whereby families are embedded in a larger social structure interconnected with other systems including the microsystem, mesosystem, exosystem, and macrosystem. Each of the four interdependent systems influence themselves and the other systems and they are influenced by other systems. Mammen and colleagues [14] describe the four sub-systems in a family ecological systems model as they related to food security:

(1) “Individual and family decisions regarding food consumption is embedded in the microsystem
(2) Interactions related to food consumption/production with extended family members, friends, and food pantries are found in the mesosystem
(3) Institutional structures, external to family and friends, that directly or indirectly influence family resources is known as the exosystem
(4) Family ideological, cultural values and beliefs, external social and economic forces that influence household food security, and supportive public policies and programs like food pantries make up the macrosystem”

Thus, the ecological perspective and family ecological systems theory are used to help explain the ways in which food security for Marshallese individuals and families is influenced by the many systems in which they interact.

### 2.2. Food Security from an Ecological Systems Model

#### 2.2.1. Demographic Characteristics

We included demographic questions in our survey to provide a snapshot of Marshallese respondent characteristics.

Race, Ethnicity, and Migrant Status. From an ecological perspective, race and ethnicity concepts are systemic determinants that interact with social, economic, and political contexts for individuals and families [15]. We know that immigrant and other non-native populations in the U.S. are more likely to experience food insecurity and to have uncertain access to foods compared with the native population. This may be due, in part, to low wages, limited education, and language barriers [16]. It is important to note that the Marshallese are considered migrants and, therefore, do not have the same legal standing of immigrant groups; as a result, they are not eligible to access important systemic supports, such as SNAP, to mitigate household economic hardships and to offset food insecurity [15,17]. Given that nearly 40% of Marshallese adults in NWA have type 2 diabetes and 32.6% have prediabetes [7,18], inconsistent access to healthy foods due to cost and/or availability has been correlated with food insecurity [19].

Education Level. Immigrant and refugee households in the U.S. are less likely to have access to opportunities for increased education and employment due to several systemic barriers that are described later in this manuscript. As a result, they are more likely to experience low wages, less secure employment, and increased vulnerability to chronic stress, thereby putting them at greater risk of food insecurity [5]. Many citizens of the Marshall Islands stop their education at the sixth grade, due to limited advanced educational opportunities in the Marshall Island atolls [20]. A recent study in NWA of Marshallese parents who participated in a local college savings program for their children found that almost 60% had a high school diploma or less [21]. Marshallese respondents indicated that their life in the U.S. would be much improved if they had increased access to the education and job training systems and if they could transfer their college credits from the Marshall Islands to local higher education institutions [20].

Employment. From an ecological perspective, understanding the ways in which the labor market system is related to hunger is important for institutionalizing initiatives to eradicate food insecurity [22]. Employment has long been linked with household income, educational achievement, and functional status [23]. For the purposes of this study, we are interested in whether employment is significantly related to food security.

Income and Poverty. The interdependent relationships between ecological, economic, and social systems and food security are multifaceted [4]. Research indicates that income poverty [24] and asset poverty are the most significant factors affecting a household’s food security [3]. Mayer and Jencks [24] and Mirowsky and Ross [25] use the terms material hardship and economic hardship, respectively, to describe the interdependence of multiple individual and family needs, including food insecurity, that influences struggles within a household’s ecological system. For most U.S. households, income is used to pay for immediate household needs for food, shelter, housing, and healthcare, while assets (i.e., savings, investments) are often relied upon during times of economic stress to offset unexpected financial crises such as unemployment, a car breaking down, or a death in the family [26]. Assets also allow individuals and families to bequeath monetary and tangible resources to their children [27]. Households without financial resources to serve as a financial buffer are less likely to withstand a financial shock [28].

#### 2.2.2. Fringe Economy as an Alternate Financial Avenue

At one time or another, many low- and moderate-income households that experienced economic strain accessed the fringe economy to help meet their financial needs [29]. The fringe economy is part of an alternate financial system that affords low- and moderate-income individuals and families financial support that is often unavailable through mainstream financial institutions [30]. The fringe economy is made up of pawn shops, check cashing stores, rent-to-own stores, payday lenders, etc., that often charge high interest rates and fees [31,32]. Families that are unbanked, have limited or no savings, or other available cash, may find no other alternative but to access the fringe economy to meet their immediate economic needs; unfortunately, high interest rates from the fringe economy often result in long-term heavily indebted consumers [30,31,32].

#### 2.2.3. Functional Status

Participation in the U.S. health care system is vital to our physical and mental health. Many research studies have found that food security is a risk factor for poor health, including diabetes, hypertension, coronary heart disease, etc. [33]. For example, the number of chronic health conditions is 18% higher for food insecure adults in the U.S. [24]. Turning to the Marshallese community, a pre-COVID-19 study found that 74% and 77% of Marshallese Islanders with diabetes in NWA declined healthcare services and had no usual source of health care, respectively, versus 15% and 7% of the U.S. diabetic population [18]. This may explain, in part, why the Marshallese life expectancy is 20 years lower than American life expectancy [34]. Other reasons for early mortality among the Marshallese include high rates of obesity, tuberculosis, heart disease, cancer, and lack of health insurance [35]. These barriers and health issues are likely related to the higher rates of adult Marshallese deaths due to COVID-19 in NWA [6]. In terms of mental health, food insecurity has been found to be associated with increased mental health disorders, including but not limited to severe and persistent mental illness, maternal depression, and stress [14,36,37].

#### 2.2.4. Health Insurance Coverage

Health insurance provides an important avenue to access healthcare services and to finance healthcare expenses [38]. Due to personal, social, economic, and other systemic barriers, 50% of Marshallese adults who live in Arkansas are uninsured [7]. It is important to note that, due to their citizenship status, Marshallese adults were not eligible to apply for Medicaid benefits in the U.S. until 2 March 2021 [39]. Turning to Medicare, Marshallese adults may be eligible for benefits if they meet U.S. employment requirements [40]. Lack of health insurance coverage is also a risk factor for food insecurity, particularly since many immigrants, refugees, and migrant groups do not seek consistent healthcare services due to cost and stigma [7,27,37].

#### 2.2.5. Economic Strain

Without adequate household income, savings, and assets, households often experience economic strain [29]. Economic or financial strain is defined as not being able to meet essential household expenses, including but not limited to the inability to pay rent or mortgage, eviction, unpaid utility, phone, healthcare, and/or dental bills, and food insecurity [41]. Economic strain caused by negative economic events such as unemployment, poverty, and debt, can influence an individual’s anxiety or feelings of not coping [28]. As a result, the systemic influence of economic strain has been associated with decreased physical and mental health, decreased coping strategies, reduced job opportunities, increased healthcare expenditures, and increased mortality [28]. Using an ecological systems lens, safety net programs such as SNAP have helped decrease household economic strain and food insecurity [41]. Unfortunately, the Marshallese are not eligible for SNAP benefits [40].

#### 2.2.6. Food Security

The sociodemographic characteristics that have been shown to influence higher rates of food insecure individuals and families are as follows: persons of color, low income and single-headed households, families with children, and households located in the southern U.S. [33]. From a cultural perspective, many food customs were transferred from the Marshall Islands to NWA upon their arrival. Namely, communal meals both with family members and friends are prioritized within the community. From a micro-systems perspective, barriers to food security are often partially attributed to some of these cultural factors. For example, the large family sizes and inaccessible food options from the Marshall Islands make maintaining a nutritionally secure diet even more difficult. Prior to the pandemic, 80% of NWA Marshallese respondents reported either low or very low food security, thus disproportionately impacting them when compared to other NWA households during the pandemic [19]. Not surprisingly, Marshallese children in NWA face food insecurity rates at a higher prevalence than those of their peers [15].

## 3. Materials and Methods

This research study consisted of cross-sectional data collected online with adult Marshallese community members in Northwest Arkansas. Data collected were designed to provide a snapshot of food security for Marshallese families during the COVID-19 pandemic as well as systemic supports and barriers to achieving food security.

### 3.1. Survey Instrument and Recruitment

We developed an online survey, comprised of standardized and non-standardized questions, for this study. Sections of this survey have been utilized in previous studies [21,30]. The survey includes 47 questions divided into six sections and takes between 15 and 20 min to complete. We offered the survey in both English and Marshallese. The first section of the online survey includes 11 questions related to demographics. In the second section, participants were asked to identify whether they engaged in any of seven fringe economy practices during the previous year, an 8th question asked if they had to pay late fees. Section Three asked participants to respond to three questions related to their functional status and one question related to the frequency with which any physical and/or mental health problems interfered with their social activities. Section Four included two questions related to health insurance coverage. In Section Five, 13 questions were asked of respondents describing some of the ways in which they had experienced economic strain as well as the frequency of their experiences. Finally, participants were asked 9 questions in Section Six related to the frequency with which they experienced food insecurity in the past month. The online survey was conducted between 14 October and 9 November 2020. Participants who completed the survey received a link to access a USD 20 Walmart Gift e-card for their time. Table 1, Table 2, Table 3, Table 4, Table 5 and Table 6 provide an overview of the questions asked of respondents in the six sections of the survey.

#### 3.1.1. Demographics

We collected data related to participant gender, age, language(s) spoken, marital status, employment status, education level, and household size.

Household Income. Annual household income was treated as a continuous measure.

Household Assets. Homeownership and savings account ownership were measured dichotomously (1 = ownership; 0 = non-ownership).

Education Level. The following four levels of education data were collected: (1) less than high school graduate, (2) high school diploma or GED, (3) some college, did not graduate, (4) graduated from college (2- or 4-year degree), and (5) graduate school.

**Table 1:** Demographic Characteristics.

Variable	Response Options
Gender	Male Female
Age	Fill in the blank
Spoken Languages	Marshallese English Other
U.S. Arrival Year	Fill in the blank
Marital Status	Never Married Married Separated Widowed Divorced
Number in Household	Fill in the blank
Highest Educational Level	Less than high school graduate High school diploma or GED Some college, did not graduate Graduated from college (2- or 4-year degree) Graduate school
Employment Status	Employed full-time or more than full-time Employed part-time Unemployed Unemployed but seeking employment
Do you currently:	Own your own home Live w/other people and not pay rent Live w/other people and contribute part of rent Rent your home/apartment Live in public housing Live in Section 8/voucher housing
Gross Annual Household Income	USD 0–4999 USD 5000–9999 USD 10,000–14,999 USD 15,000–19,999 USD 20,000–24,999 USD 25,000–29,999 USD 30,000–34,999 USD 35,000 and up
Savings Account for Self or Child(ren)?	Yes No

#### 3.1.2. Fringe Economy Scale

We developed a non-standardized, 7-item, dichotomous scale to capture the number of times respondents used the fringe economy in the previous year. Fringe economy participation included the questions embedded in Table 2. Cronbach’s alpha reliability coefficient for the scale is 0.78.

**Table 2:** Fringe Economy Participation.

Questions	Response	
Have you sold or placed on hold an item at a pawn shop?	Yes	No
Have you rented any household items at a rent-to-own store?	Yes	No
Have you cashed a check at a check cashing store?	Yes	No
Have you received any funds from a non-banking financial institution?	Yes	No
Do you have a credit card with an interest rate that is over 12%?	Yes	No
Have you taken out a loan with an interest rate over 9%?	Yes	No
Have you been late in paying a credit card bill or loan?	Yes	No
If you responded yes to #23, have you had to pay late fees?	Yes	No

#### 3.1.3. Functional Status Scale

We used a shortened version of the SF-12v2 scale to measure the perceived physical and emotional health of Marshallese respondents. The larger SF-12v2 is a 12-item Likert scale with summated ratings that measure eight health concepts of functional status. The standardized scale has proven reliability and validity, as evidenced by the test–retest correlations of 0.89 and 0.76 for the physical and mental component summaries, respectively [42]. Table 3 provides an overview of our questions.

**Table 3:** Functional Status.

Questions	Response Options				
Have you felt calm and peaceful?	All the time	Most of the time	Some of the time	A little of the time	None of the time
Did you have a lot of energy?	All the time	Most of the time	Some of the time	A little of the time	None of the time
Have you felt downhearted and depressed?	All the time	Most of the time	Some of the time	A little of the time	None of the time
During the past 4 weeks, how much of the time has your physical health or emotional problems interfered with your social activities?	All the time	Most of the time	Some of the time	A little of the time	None of the time

#### 3.1.4. Health Insurance Coverage

As highlighted in Table 4, respondents were asked to indicate their current health insurance status and, if uninsured, to provide reasons for this status.

**Table 4:** Health Insurance Coverage.

Questions	Response Options
Could you please choose the best response to describe the type of health insurance, if any, you currently have?	Private health insurance through an employer or spouse/partner employer Health insurance through Medicaid or Medicare Insured through the Affordable Care Act No health insurance currently Insured through other means (e.g., the purchase of private health insurance)
If you are not currently insured, which of the following reasons best describes why you are uninsured?	Unemployed Cannot afford insurance through the workplace Employer does not offer health insurance Not eligible for Medicaid or Medicare Not needed/not worth the money or effort Employment waiting period

#### 3.1.5. Economic Strain Scale

We chose to examine household economic strain since low-resource families often struggle with food security [43]. We used a shortened version of the standardized 13-item Family Economic Strain Scale (FESS) to measure household economic strain. Higher scores on the Likert scale indicate increased economic strain. Cronbach’s alpha for the scales is 0.90 and construct validity has been demonstrated [44].

**Table 5:** Economic Strain.

Statement	Never	Seldom	Sometimes	Usually	Almost Always
In general, it is hard for me and my family to live on our present income.	1	2	3	4	5
I experience money problems.	1	2	3	4	5
Financial problems interfere with my work and daily routine.	1	2	3	4	5
I worry about financial matters.	1	2	3	4	5
Financial problems interfere with my relationships with other people.	1	2	3	4	5
I worry about disappointing my children because I can’t give them things they want.	1	2	3	4	5
I worry about having money to celebrate holidays and other special occasions.	1	2	3	4	5
I put off family activities (vacations, movies, or special events) because of the expense.	1	2	3	4	5
I feel frustrated because I can’t afford the education or training I need to get to get ahead.	1	2	3	4	5
I must put off getting medical care for family members because of the expense.	1	2	3	4	5
I must put off getting dental care for family members because of the expense.	1	2	3	4	5
I feel bad that I can’t afford to buy my children brand name clothing that other children their age are wearing.	1	2	3	4	5

Compared with other families in the U.S., would you say your income is: 1. far below average; 2. below average; 3. Average; 4. above average; 5. far above average.

#### 3.1.6. Food Security Survey

Food security was measured using the 9-item, standardized, USDA Food Security Survey [45]. The Likert-type questions in the survey captured frequency of behaviors, such as worrying about food costs, buying less expensive food, and limiting food intake during the previous month. Response options were 0–2, where zero denotes “Never”, 1 signifies as “Sometimes” and 2 represents “A lot”. We summed the scores for each respondent and then categorized them into different levels of food security. Internal validity was found to be adequate.

**Table 6:** Food Security.

Statement	A Lot	Sometimes	Never
Did you worry that food at home would run out before you got money to buy more?	2	1	0
Did the food that you bought run out, and you didn’t have money to get more?	2	1	0
Did your meals only include a few kinds of cheap foods because you were running out of money to buy food?	2	1	0
How often were you not able to eat a balanced meal because you didn’t have enough money?	2	1	0
Did you have to eat less because you didn’t have enough money to buy food?	2	1	0
Has the size of your meals been cut because you didn’t have enough money for food?	2	1	0
Did you have to skip a meal because you didn’t have enough money for food?	2	1	0
Were you hungry but didn’t eat because you didn’t have enough food?	2	1	0
Did you not eat for a whole day because you didn’t have enough money for food?	2	1	0

### 3.2. Sampling

Based on our financial resources to conduct this study, our original goal was to collect data from 150 Marshallese individuals. We collaborated with the primary social service agency that serves the Marshallese community in NWA; however, since it was closed to the public during the data collection period due to the pandemic, we were unable to recruit participants for our study via flyers, face-to-face community convenings, etc. The agency agreed that one of their staff members would contact all their adult clients via email to invite them to participate in this study. In the end, however, the staff member experienced multiple family illnesses and deaths due to COVID-19 after beginning the recruitment and data collection process and was unable to continue with recruitment after one month into the study. All other staff members were either tending to ill loved ones or focused on procuring donations and allocating groceries for the new Marshallese food pantry and were unable to assist with our recruitment. As a result, we collected data from less than half of our intended sample size.

While resource constraints limit the amount of collected data, there is often a trade-off between the costs of data collection and the value of having access to the data [46]. According to Lakens [46], even limited data are valuable in that they provide researchers with more knowledge about the research question, even when findings are not generalizable to the larger population.

Due to barriers to face-to-face data collection, we used nonprobability convenience sampling to conduct an online cross-sectional survey to explore food security and food security risk factors for Marshallese adults who reside in NWA during the first several months (14 October–9 November 2020) of the COVID-19 pandemic. Probability sampling strategies use a form of random selection while nonprobability convenience sampling is often used within developmental science to access a sample of participants that is conveniently accessible [47,48]. Many researchers used convenience sampling during the COVID-19 pandemic to procure a quick overview of the effects of the pandemic on individual, household, and community health and well-being [49,50]. Often, the reasons for this were to identify areas of need for the most vulnerable populations so that supportive services and resources could be provided in real time [51]. Some advantages to using convenience sampling include cost, usefulness for pilot studies, and the ability to collect data in a short period of time; the primary disadvantages include high sampling error [52] and a rush to effect policy and practice when generalizability to the larger population is not appropriate [51]. While convenience sampling results in less clear generalizability than probability sampling, homogeneous samples such as the Marshallese community in NWA have greater generalizability when compared with conventional convenience samples [48].

### 3.3. Data Analysis Process

Survey results were transferred to a Qualtrics database and then imported into R, an open-source statistical computing platform, where data analyses were performed. By order, we conducted descriptive, correlation, and multinomial logistic regression analyses for this study. We chose the commonly used significance level of alpha = 0.05 as our default threshold of hypothesis testing and model fitting process. To elaborate, first, we conducted descriptive analyses on all possible factors listed in our survey, which provided us with a snapshot of distributions on all assumed determinants of our target variable (i.e., food security). Pairwise correlation analyses were conducted; then multinomial logistic regression was fitted to detect true relationships among our assumed factors and dependent variable (food security). Findings from our analyses indicated that food security was significantly associated with education and economic strain. For verification purposes, we conducted Spearman’s rank correlation analyses on education vs. food security, and economic strain vs. food security; the results supported our conclusion. Finally, a variance inflation factor (VIF) score was calculated on our fitted logistic regression model to make sure there was no strong multicollinearity in our model, which would weaken the statistical power of our regression model. The resulting VIF score (<5) indicated that a moderate multicollinearity issue exists; however, it was not strong enough to require further attention.

## 4. Results

### 4.1. Descriptive Analysis of Demographic Characteristics

Age. As shown in Table 7, the age ranges of Marshallese respondents span from 18–67 years, with an average age of 33.9 years (SD = 8.95); most respondents (94.3%) are under 49 years old.

Gender. The majority (77.5%) of respondents are female; with the ratio of female to male respondents at approximately 3:1.

Language. Almost half (46.5%) of our sample report speaking only Marshallese and half (50.7%) speak both English and Marshallese. A small portion (2.8%) of respondents only speak English.

U.S. Arrival Year. Marshallese respondents report arrival to the U.S between 1987 and 2019. U.S. arrivals cluster between 2014 and 2016, during which more Marshallese respondents (24%) arrived; the remaining arrivals are evenly distributed throughout the other years.

Marital Status. Table 8 shows that almost half (47.9%) of the sample report being single, never married, and 43.7% indicate being married. There was a small number (8.4%) of separated and divorced respondents.

Education. Table 9 shows that approximately one third of respondents do not have a high school diploma, one third graduated from high school, and one third attended and/or graduated from a two- or four-year college. No respondents report attending graduate school.

Employment and Household Income. Turning to the relationship between employment and household income, Table 10 highlights that no matter what the employment status of respondents, the majority of sampled Marshallese (84.5%) live at very low (under USD 20,000) annual household income levels.

Household Assets. In terms of household assets, the majority (70.4%) of Marshallese respondents do not own a house and 85.9% do not have a saving account.

#### 4.1.1. Fringe Economy Findings

We developed a Likert scale to measure the frequency in which Marshallese respondents used the fringe economy in the past 12 months. The scale consists of eight items; each item has two options as 0 means “no use” and 1 means “use”. We summed up all scores of each item of a respondent to obtain an overall score. The range of each respondent is on (0, 8). On average, Marshallese respondents used one to two fringe economy services ($\bar{x}$ = 1.26) within the past year.

#### 4.1.2. Functional Status Findings

Functional status was measured using four Likert items which address specific physical and mental health questions in the past four weeks. As shown in Table 11, on average, most (95.8%) respondents note feeling calm, peaceful ($\bar{x}$ = 2.77), and energetic ($\bar{x}$ = 2.87) “most of the time.” Paradoxically, 73% of respondents indicate that they feel downhearted and depressed ($\bar{x}$ = 1.42) either “a little” or “some” of the time and 8% feel this way most or all the time. Finally, 48 of 71 (68%) respondents report that their emotional problems interfere with their social activities to some extent.

#### 4.1.3. Health Insurance Coverage Findings

Table 12 shows that 88% of respondents are employed either full-time or part-time. Among full-time employees, 56.2% have insurance from their employers; for the unemployed group, 71.4% do not have health insurance coverage.

#### 4.1.4. Economic Strain Findings

We scored economic strain by summing the first 12 Likert items, resulting in a mean score for each individual observation. Economic strain mean score ranges (0, 3.67) with a mean of 1.41 (sd = 0.76) for all observations. This means that most Marshallese respondents experience economic strain (between “Seldom” and “Sometimes”) on all items. In addition, 28.2% of respondents reported experiencing economic strain “sometimes” or “frequently.” We then examined respondents’ economic strain status for each of the 12 Likert scale items to gain insight into the severity of economic strain experienced. Mean item scores range from 0.93 to 2.04, with the least severe item noting “I must put off getting medical care for family members because of the expense” and the most severe item indicating “I experience money problems.”

An additional question asked respondents to compare their household income with other families in the U.S. The response scale was between 1 and 5, where 1 denotes as “far below average” and 5 denotes as “far above average”. Findings show that almost half (46.5%) of respondents feel that their household income is on par with the national average and almost half (49.3%) view their incomes to be below and far below the national average.

#### 4.1.5. Food Security Findings

As indicated in Table 13, 81.7% of Marshallese respondents reported low or very low food secure households in the past month, almost 9% reported marginal food security, and 10% had not experienced any limitations in terms of food security.

### 4.2. Correlation Analyses

Household Income vs. Food Security Levels. We examined whether food security and household income are correlated, with the assumption that higher household incomes are associated with higher rates of food security. While we found some relationships between the two variables, the results of Spearman’s rank correlation indicated no statistically significant relationship between them (rho = 0.12, *p*-value = 0.32).

Model Fitting. First, we fit a multinomial logistic regression on food security with all possible explanatory variables to examine which variables are correlated with food security. Findings in Table 14 indicate that only education and economic strain have significant effects on food security levels. Increased education levels are correlated with increased food security for participants. Not surprisingly, increased household economic strain is correlated with decreased household food security. We also examined the collinearity among predictor variables—while there is moderate correlation between predictor variables in the model, it is not strong enough to require attention.

To verify our results, we conducted a Spearman’s rank correlation on education vs. food security, and economic strain vs. food security. The rho scores and *p* values confirm that both associations are statistically significant. In other words, there is a moderate negative association (rho = 0.4) between education and food security scores, and a moderate positive association between economic strain and food security scores (rho = 0.61). Thus, as education level increases, food security increases and as economic strain increases, food security decreases.

### 4.3. Limitations

It is important to note several limitations of this study. First, we used a nonprobability convenience sample to recruit study participants through the primary local Marshallese social service agency. While the agency has a far reach into the Marshallese community, we likely missed accessing many members of the Marshallese community who either do not have a relationship with the agency or who do not have easy access to a smart phone, computer, and/or the Internet. Thus, it is important to note that our sample may not be representative of the Marshallese community in NWA. Future research studies that use a randomized sample of Marshallese adults in NWA are recommended.

A second limitation is the small sample size of this study. Given that we had a limited budget for this project, and that recruitment was halted earlier than anticipated due to COVID-19 illnesses and deaths among staff, we were not able to reach our goal of 150 Marshallese adults in our study. Future research that includes larger samples will allow research findings to be more representative of and generalizable to the entire Marshallese community.

A third limitation is that this cross-sectional study explores food insecurity at one point in time; as a result, we may not have captured an accurate picture of food security for Marshallese households over time. Future research that is longitudinal in nature may show different results for Marshallese households in NWA.

Finally, it is important to note that during the data collection phase of this study, members of the Marshallese community informally allocated food donations to Marshallese families in need. Unfortunately, we do not have data on whether respondents in our study received food donations. Thus, it may be that our results do not reflect the full effect of COVID-19 on food security for our Marshallese sample.

## 5. Discussion

During the fall of 2020, we collaborated with the primary NWA Marshallese social service agency for this study. Given that the agency was closed to the public due to COVID-19, we decided to engage in an online study. For our pilot study, we applied family ecological systems theory and the ecological perspective [13,14] to better understand food security for a small sample of Marshallese individuals and households in NWA during the first year of the COVID-19 pandemic. The ecological framework allows us to view the ways in which the systems with which Marshallese families interact influence household food security. We found Marshallese households in NWA experienced many systemic (i.e., personal, social, economic, and health) challenges during COVID-19 in 2020 that were either directly or indirectly related to food security.

Using a systems framework, we also found that the pandemic emphasized health disparities as well as problems with healthcare equity and access, resulting in dire outcomes for the Marshallese community. It is important to note that the Marshallese community was experiencing a health crisis at much higher rates than non-Hispanic white and other communities of color during the time we collected data. For instance, while the Marshallese comprise only 3% of the NWA population, they accounted for nearly 20% of all COVID-19 cases and 38% of all COVID deaths; this rate was 25 times higher than the rate of death for the Hispanic community [6].

Due to Marshallese community member COVID-19 illnesses and deaths, our data collection was halted before we were able to recruit our intended sample size. While our small sample size precludes us from generalizing our results to the larger NWA Marshallese population, we believe that the information collected is meaningful in that it provides us with information not yet collected from the NWA Marshallese community, particularly as it relates to individual and household measures of well-being and systemic barriers to achieving food security. Thus, the high rates of income and asset poverty in the Marshallese community, ineligibility for SNAP food stamps for Marshallese individuals born in the Marshall Islands, poor job conditions, high rates of disease and health disparities, as well as linguistic and cultural factors all contributed to the disproportionately high COVID-19 illness and death rates of the NWA Marshallese in comparison with Latinos and all other Arkansas communities [6,8,10,11].

### 5.1. Demographics

Most of our demographic variables (e.g., age, race/ethnicity, marital status, etc.) were not significantly associated with food security. However, we did find a significant relationship between food security and education level. While they did not achieve statistical significance, we feel that household income and asset poverty findings are meaningful, so we present them in more detail here.

Income Poverty. Although we did not find a significant association between household income and food security, it is worth noting that the overwhelming majority of Marshallese respondents live in poverty, regardless of employment status. For example, 85% of respondents report ≤ USD 19,999 in annual household income, 11% report ≤ USD 34,999 and 4% report annual household incomes over USD 35,000. Given the negative effects of material hardship on food security [24,25,53], in the future, it is important to explore initiatives that increase household income for Marshallese households.

Asset Poverty. In addition to income poverty, most Marshallese respondents are asset poor. A small, dedicated savings pilot program in the Marshallese community was recently concluded by the first and third authors of this current study—the program was designed to support homeownership, small business development, post-secondary education, and vehicle ownership for low- and moderate-income Marshallese households through a savings match. An additional small, dedicated emergency savings program for the Marshallese community was initiated by the authors and a national funder during the pandemic. Given that the pandemic created many financial crises for Marshallese program participants, funders approved adding the use of funds to help offset financial crises due to illness, job loss, and death. Twelve of the twenty-five participants who completed the former dedicated savings program, used some matching funds toward rental assistance. In terms of the latter dedicated emergency savings program, participants used emergency funds on rental assistance, car repairs/payments, food, funeral expenses, and utilities. Thus, the lack of household assets and the need for additional financial support during COVID-19 for Marshallese community members reflects previous research whereby low-income and low-asset households are often ill-equipped or unable to weather financial shocks [26,28]. Similar ‘safety net’ programs that help Marshallese households build household income and wealth are worthy of attention and resources in the future.

Education. Educational level was found to be significantly correlated with food security for Marshallese respondents. We found that over two-thirds of Marshallese respondents have a high school diploma or less; these findings support previous research on educational attainment for the Marshallese in Northwest Arkansas [20,21]. Low education levels also help explain corresponding low incomes and may, indirectly, help explain the high rates of food insecurity. From an ecological systems perspective, respondents in our study may have faced similar systemic educational barriers shared by NWA Marshallese adults in previous studies, particularly as they relate to problems increasing human capital (e.g., transferring course credits from Marshall Island universities to NWA universities) as well as difficulties showing adequate documentation and proof of legal identity for enrollment in higher education [20].

Based on our results, the post-secondary educational system seems to serve as a buffer for household food insecurity. While historically, the NWA higher education community has not dedicated resources to the recruitment and retention of Marshallese students, within the past two years the University of Arkansas has invested in community- and family-based initiatives to attract prospective Marshallese students. Future efforts to create a less difficult pathway for the Marshallese to access the post-secondary educational system and, thereby, increase educational levels could, in turn, positively influence food security for Marshallese households. In other words, culturally appropriate, systemic efforts, whereby the local higher education institutions collaborate with NWA Marshallese social service and education agencies to recruit and enroll new and transfer students locally and from the Marshall Islands, would be helpful in increasing educational attainment and building additional employment skills [20].

Employment. While we did not find a direct, significant relationship between employment and food security, several meaningful findings are worth consideration. For example, descriptive findings indicate that Marshallese respondents are as likely to be employed as unemployed. In addition, no matter what the employment status, Marshallese respondents report very low household incomes. As we discussed previously, the NWA Marshallese face many systemic barriers to workforce development and educational attainment. Poverty reduction initiatives designed to invest in productive and remunerative employment opportunities for low-income Marshallese households may help improve employment status and job skills, increase household financial resources, and reduce food insecurity [22].

### 5.2. Fringe Economy—Future Strategies

Findings indicate that most Marshallese respondents participated in fringe economy financial systems at some point during the previous 12 months to meet their financial needs. Fringe economy participation places families at increased risk of long-term debt and serves as a barrier for increasing household income and assets [32]. Efforts where mainstream financial institutions reach out to the Marshallese unbanked or underbanked households and programs that provide financial education about debt and high interest rate consequences in the U.S. may help offset long-term debt inherent in the fringe economy and pave the way for household asset development [30,31].

### 5.3. Functional Status—Future Strategies

The healthcare system is integral to providing physical and mental health services for U.S. residents. The relationship between food security and health and mental health status is well documented in the literature [33,36]. While our research did not find a statistically significant association between food security and health/mental health outcomes and health insurance coverage, we did find high rates of food insecurity and 75% of respondents noted some measure of depression and/or feeling downhearted. In addition, more systemic work is needed locally to encourage Marshallese adults to enroll in Medicaid and Medicare, when applicable, to meet their healthcare preventative and treatment needs. In terms of systemic barriers, Marshallese individuals may not be aware of the mental health supports that are available in their community or do not wish to access them due to stigma. In future research, it would be beneficial to collect more detailed health data from a larger sample to better understand the relationships between functional status and food security for the Marshallese community.

### 5.4. Health Insurance Coverage Discussion

In 2020, 8.6% of people in the U.S. were uninsured [38], compared with 50% of Marshallese respondents in our study. These findings reflect previous research on Marshallese health insurance coverage [7]; the low rates of health insurance coverage are particularly concerning given the high rates of chronic disease within the Marshallese community, which were compounded during COVID-19 [54]. For example, according to the CDC [6], the lack of health insurance coverage likely played an important role in the disproportionate number of COVID-19-related illnesses and deaths within the NWA Marshallese community.

### 5.5. Economic Strain—Future Strategies

Our findings suggest that most Marshallese households experience economic strain, also known as financial and material hardship, to some degree. We also found that economic strain and food security are significantly associated. This supports previous research on the negative influence of household economic strain on household measures of well-being, including food security [41]. Safety net programs like SNAP have proven to provide an important buffer to food insecurity for U.S. households, particularly in communities of color [40,41]. Given that Marshallese families with children are not eligible to receive SNAP benefits, future policy change efforts to allow for SNAP participation in Arkansas and nationwide is highly warranted.

### 5.6. Food Security Discussion

The NWA Marshallese community had high rates of food insecurity rates pre-COVID-19, so it was not surprising that rates of food insecurity increased during the pandemic. Findings from our small study indicate that, even when controlling for sociodemographic risk factors, self-reported food insecurity rates were high for Marshallese respondents during the fall of 2020 when the COVID-19 pandemic was well under way. Using an ecological systems perspective, this finding supports previous research that inadequate access to culturally appropriate food choices, difficulties with community food deserts, and limited household income all contribute to food insecurity for immigrant and refugee communities [1]. Even with short-term strategies, such as the newly initiated Marshallese food pantry, Marshallese household food insecurity rates were very high during the early months of the pandemic for our sample. Additionally, while community pantries and churches serve immediate food needs, these programs do not reflect long-term solutions for combating food insecurity in Marshallese populations [19]. Our findings support previous research, suggesting that long-term solutions in the NWA Marshallese community are necessary [19].

### 5.7. Implications

We can draw several policy, research, and practice implications from this study. Our findings indicate that economic strain and education are significantly associated with food security for Marshallese households. Given the low education levels of Marshallese adults in NWA, strategies to help overcome administrative barriers (e.g., migrant status documentation, transfer credits from Marshall Island universities) to post-secondary education enrollment would benefit the Marshallese. These strategies also have the potential to increase household income and assets, decrease food insecurity, and improve global workforce readiness among Marshallese employees. Findings demonstrated a rather confusing snapshot of functional status for Marshallese respondents. Namely, respondents reported feeling calm, peaceful, and energetic while also noting feelings of depression at times. Future research that can define more comprehensively the perceptions of Marshallese respondents for physical and mental health conditions can provide a better overview of functional status. Finally, efforts to enact policy in Arkansas whereby the Marshallese are eligible for SNAP benefits have the potential to increase food security and decrease health disparities for Marshallese community members. Pre-COVID and COVID rates of food insecurity among the NWA Marshallese were extremely high; addressing hunger needs for the Marshallese community is crucial for increasing personal, social, and economic well-being.

## 6. Conclusions 

Our small cross-sectional pilot study highlighted high rates of food insecurity for Marshallese households during COVID-19. The fact that those born in the Marshall Islands are unable to qualify for SNAP benefits places them at increased risk for food insecurity. As a result of food insecurity in the community, the Arkansas Coalition of Marshallese (ACOM) social service agency applied for and received approval to build a community food pantry for Marshallese community members. The Enrã Food Pantry was launched on 26 August 2020 [55]. Regardless of whether study respondents accessed the Marshallese food pantry during our data collection phase, food insecurity rates were still very high in the community. A 2022 interview with Melisa Laelan, executive director of ACOM, revealed the number of Marshallese served by the pantry has increased steadily since its inception; currently, the pantry is open once a week and serves 70 families/420 individuals per week [56]. The pantry helps to support an urgent need in the Marshallese community but increased community-based practices and innovative agriculture and food science research warrant consideration in addressing food insecurity and healthy eating practices in the Marshallese community.

As a collective community, community-based approaches that center the Marshallese population as cultural experts in collaboration with non-profit agencies, philanthropic funders, healthcare providers, and researchers are welcomed and are often successful in addressing needs within the Marshallese community [56]. In terms of community-based approaches, educational and public health efforts that promote awareness of preparing healthy food options that remain reminiscent of Marshallese culture would be beneficial. It is also important that such efforts include strategies to ensure readily accessible ingredients to prepare healthy, yet culturally responsive alternatives within the Marshallese community. While many Asian markets carry such ingredients, the markets are often outside of the Marshallese community and difficult to access without reliable transportation. Philanthropic and venture capital efforts would make great partners in eliminating the food deserts within the Marshallese community. In addition, the many advances in food science and agriculture research may also play a major role in addressing challenges such as access to the healthier food option indigenous to the Marshall Islands by using advances in food science to grow such produce in the NWA area.

## Figures and Tables

**Table 7:** Respondent Age Categories.

Age Groups	Frequency	Percentage
18–35 yrs.	4	57.7
36–48 yrs.	26	36.6
49–55 yrs.	3	4.2
≥56 yrs.	1	1.4

**Table 8:** Marital Status of Marshallese.

Marital Status	Frequency	Percentage
Never Married	34	47.9
Married	31	43.7
Separated	5	7.0
Widowed	0	0.0
Divorced	1	1.4

**Table 9:** Distribution of Education Levels.

Education	Frequency	Percentage
Less than High School	23	32.4
High School	26	36.6
Some College	10	14.1
Graduated 2- or 4-Year College	12	16.9

**Table 10:** Contingency Table of Employment and Household Income.

	USD 0–USD 19,999	USD 20,000–USD 34,999	USD 35,000 and up
Employed FT	28	1	3
Employed PT	10	1	0
Unemployed	22	6	0

**Table 11:** Functional Status.

	Calm and Peaceful	Energetic	Depressed	Total
None of the time	2	2	19	23
A little of the time	3	4	11	18
Some of the time	25	19	35	79
Most of the time	19	22	4	45
All of the time	21	24	2	47
Not Answered	1	0	0	1
Total	71	71	71	-

**Table 12:** Contingency Table of Employment and Insurance.

	Employer Insurance	Medicaid/Medicare	Affordable Care Act	No Insurance	Other Insurance	Total
Employed FT	18	3	1	10	0	32
Employed PT	4	3	0	4	0	11
Unemployed	3	4	0	20	1	28
Total	25	10	1	34	1	71

**Table 13:** Distribution of Food Security Levels.

Food Security Levels	Frequency	Percentage
High food security	7	9.86
Marginal food security	6	8.45
Low food security	29	40.85
Very low food security	29	40.85

**Table 14:** Output of Ordinal Logistic Regression.

Variables	Value	Std. Error	t Value	*p* Value
Household	−0.021	0.012	−1.66	0.097
Education2	−0.172	0.651	−0.264	0.792
Education3	−1.764	0.817	−2.16	0.031 *
Education4	−2.002	0.861	−2.325	0.02 *
FRINGE_sum	−0.007	0.008	−0.795	0.427
HEALTH_sum	−0.066	0.044	−1.497	0.135
ESTRAIN_sum	0.127	0.036	3.48	0.001 *
HealthInsurance2	0.069	0.825	0.084	0.933
HealthInsurance3	1.282	2.05	0.625	0.532
HealthInsurance4	−0.197	0.57	−0.345	0.73
HealthInsurance5	−1.118	1.814	−0.616	0.538
High_food_security|Marginal_food_security	−2.482	1.06	−2.342	0.019 *
Marginal_food_security|Low_food_security	−1.465	1.006	−1.456	0.145
Low_food_security|Very_low_food_security	1.549	1.012	1.53	0.126

* p = 0.05.

## Data Availability

The data are not publicly available since the participants of this study did not give written consent for their data to be shared publicly, and, given the small sample size and small Marshallese community, study participants could be identified.
